# Text Semantic Classification of Long Discourses Based on Neural Networks with Improved Focal Loss

**DOI:** 10.1155/2021/8845362

**Published:** 2021-01-07

**Authors:** Dan Jiang, Jin He

**Affiliations:** ^1^School of Computer Science, Beijing University of Posts and Telecommunications, Beijing 100876, China; ^2^Key Laboratory of Trustworthy Distributed Computing and Service (BUPT), Ministry of Education, Beijing 100876, China

## Abstract

Semantic classification of Chinese long discourses is an important and challenging task. Discourse text is high-dimensional and sparse. Furthermore, when the number of classes of dataset is large, the data distribution will be seriously imbalanced. In solving these problems, we propose a novel end-to-end model called CRAFL, which is based on the convolutional layer with attention mechanism, recurrent neural networks, and improved focal loss function. First, the residual network (ResNet) extracts phrase semantic representations from word embedding vectors and reduces the dimensionality of the input matrix. Then, the attention mechanism differentiates the focus on the output of ResNet, and the long short-term memory layer learns the features of the sequences. Lastly but most significantly, we apply an improved focal loss function to mitigate the problem of data class imbalance. Our model is compared with other state-of-the-art models on the long discourse dataset, and CRAFL model has proven be more efficient for this task.

## 1. Introduction

The semantic classification of long discourses refers to the extraction of modus operandi features from textual information. Essentially, it is a special discourse classification task. Many kinds of significant information, such as time and place of event, person, and type of event, are included in description text. Among these, the time, place, and person of an event can be extracted by entity recognition. However, the type of event cannot be obtained by entity recognition; instead, they can be derived from semantic comprehension. In this task, extracting the intrinsic semantic feature of the discourse by applying the deep learning approach is necessary [1].

In recent years, convolutional neural networks (CNNs) and recurrent neural networks (RNNs) have been widely applied to tasks of text classification [2–6]. CNNs are able to capture local features from spatial data. In contrast to CNNs, RNNs are effective in processing sequence information. Long short-term memory (LSTM) [7], as a developmental architecture of RNN, can solve gradient vanishing and explosion problems of long text sequence learning. Researchers have presented combinations of LSTM and other methods [8–11] to improve the performance of LSTM in text classification.

The semantic classification of long discourses is a challenging task. First, the text vector of long discourses is high-dimensional. This high-dimensional input increases the number of parameters and renders it difficult to optimize in RNN model. This problem can be solved by the dimensionality reduction of CNN. Second, a long discourse text has the problem of sparsity. A discourse text usually comprises 500 to 2000 words, and only a few of them are useful in the classification task. Neural network cannot focus on important information when it learns text features, but the attention mechanism can help solve this problem effectively [12]. Finally, a long discourse text involves large number of classes. Generally, the number of classes of a text classification task does not exceed 20. Nevertheless, if a discourse text has more than 200 classes, the problem of class imbalance will be serious. Focal loss enables the training of highly accurate dense object detectors when the image data are imbalanced in terms of class [13]. We can transform this powerful loss function into the text classification task to solve class imbalance problems.

For event extraction of long discourse, we propose a deep sequence model based on the residual network (ResNet) with attention mechanism, bidirectional LSTM (BiLSTM), and improved focal loss function. The main contributions of our model are firstly applying ResNet for semantic analysis of long discourse and improving focal loss function with adding Gaussian weight to address the class imbalance issue. We evaluate the effectiveness of CRAFL and compare our results with a wide range of baselines. Experiments show that CRAFL performs better than baselines in the task of semantic classification of long discourses.

## 2. Related Work

### 2.1. Deep Learning Models for Discourse Classification

Deep learning has superior performance in text classification task. In various RNN structures, LSTM is one of the most powerful networks for text sequence processing. Huang et al. [14] presented a LSTM-based approach to model long texts and exploited the semantic relations between sentences in document-level sentiment classification. This model outperformed several variants of LSTM on three publicly document-level review datasets. Yan et al. [15] introduced two kinds of combinations of LSTM networks for document representation and multilabel ranking. The labels of documents were represented as a semantic tree that can capture the correlations between labels.

ResNet [16] outperformed the other models in the ImageNet Large Scale Visual Recognition Challenge in 2015. ResNet can train deep networks more quickly than the traditional CNN, and it can reduce gradient explosion and disappearance. Researchers have attempted to apply ResNet in nature language processing to capture the internal semantic information of texts. Zhang et al. [17] proposed an attention-based ResNet model to recognize medical concept relations in Chinese electronic medical records. In this model, ResNet can reduce the negative impact of corpus noise to parameter learning. Hu et al. [18] presented a sentiment classifier that combines ResNet and attention mechanism. This model achieved state-of-the-art performance on three public Chinese sentiment classification datasets.

However, both CNNs and RNNs have some problems in dealing with long texts. CNNs lack the ability to learn sequential correlations, while RNNs may encounter a sharp increase of parameters when processing high-dimensional vectors. In solving these problems, researchers have combined RNNs with CNNs or other structures. Yoon and Kim [8] introduced a multichannel lexicon integrated CNN-BiLSTM model for sentiment analysis. The model can capture both long-term dependency and high-level features of short texts. Chen et al. [6] proposed a sentiment analyzer by using BiLSTM, conditional random fields, and CNN. The opinionated sentences are classified into three types, and each group of sentences was separately fed into the CNN layer for sentiment classification.

The combination of LSTM and attention mechanism has been used successfully to resolve problems of long sequences. You et al. [9] proposed an extreme multilabel text classification model based on BiLSTM and the multilabel attention mechanism. This method outperformed RNN and CNN in five benchmark datasets. Zhang et al. [10] presented the coordinated CNN-LSTM-attention (CCLA) model to learn the semantic and emotional information of a document. The model not only captured local semantic information within sentences but also obtained the joint meaning produced by sentences. Liu and Guo [11] introduced an architecture called attention-based BiLSTM with convolution layer (AC-BiLSTM) similar to Zhang's method. However, compared with Zhang's work, Liu's model has lower time and space complexities and it also gained good results.

Overall, many studies based on the combination of LSTM and other structures have been conducted and they achieved outstanding performances in text semantic classification. These studies serve as the basis of CRAFL.

### 2.2. Solutions to Imbalance Data

Imbalanced data distribution is one of the challenges in text classification, and many researchers have endeavored to solve this problem. The solutions include oversampling [19, 20], weighting class [21, 22], and so on. Li et al. [19] presented an oversampling technique by directly creating synthetic texts. This approach addressed the imbalanced data problem in the sentiment classification task. Liu et al. [21] proposed the gravitation model to alleviate the class-imbalanced problem by learning different weighted factors for various classes, which led to a Voronoi partition. Pouramini et al. [23] introduced a probabilistic feature selection model for two-class imbalanced text data.

Focal loss was proposed to address the class imbalance issue in object detection tasks by reshaping the standard cross entropy loss [13]. Focal loss is superior not only in the computer vision field but also in the text mining domain. Sun et al. [24] proposed an information extraction model to analyze biomedical literature and applied an improved focal loss function to mitigate class imbalance. The focal loss function was proven effective in dealing with class imbalanced texts.

## 3. Model

**Figure 1:**
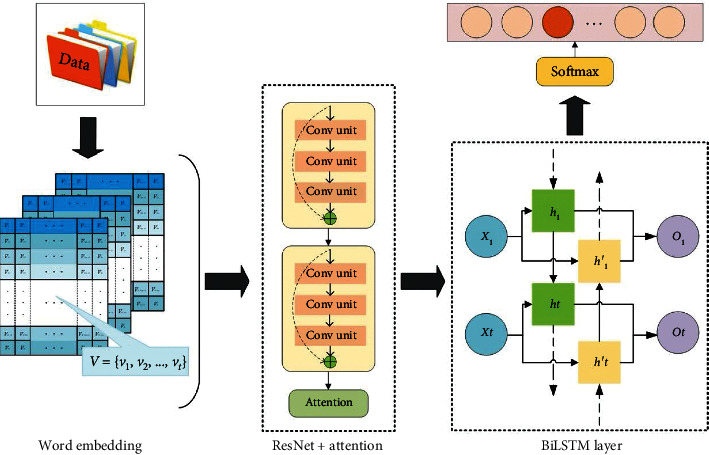
The framework of CRAFL method.

The framework of CRAFL consists of four parts: word embedding layer, CNN layers, BiLSTM layer, and output layer. The architecture of CRAFL model is shown in Figure 1.

First, when a text was inputted, the word embedding layer transfers this text into a discourse vector by using a dictionary index. Second, the CNN layers with attention mechanism are used to extract the features of the text, and the BiLSTM layer learns the features. Finally, the model outputs the classification result through the output layer by using softmax and improved loss function. The details of each layer are described in succeeding sections.

### 3.1. Word Embedding

The core idea of word embedding is mapping words to real vectors. Before word embedding, the text needs to be preprocessed to gain data structural expression. The word preprocessing includes removing low-frequency words, removing stop words, and Chinese segmentation. In particular, our method utilizes Jieba, one of the most widely used tools for Chinese word segmentation, to transfer text into an array of words. In the sequence {*w*_1_, *w*_2_,…, *w*_*l*_}, *l* is the length of input text. We used Word2vec [25], a toolkit Google launched in 2013, to acquire the word vector matrix *V*={*v*_1_, *v*_2_,…, *v*_*l*_}, *V* ∈ *R*^*l*×*d*^*w*^^, where *d*^*w*^ is the size of word vectors.

Words have different meanings in different contexts, and thus we fine tune the word vector for each discourse during training to improve the performance of features extraction. We applied the same dataset as that used in this study as the dictionary index of the Word2vec model.

### 3.2. ResNet with Attention

**Figure 2:**
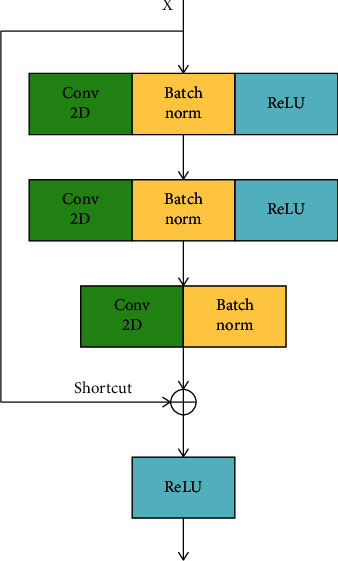
The architecture of the ResNet block.

CNN is one of the most commonly used connectionism models for feature extraction. In the convolution layer, connectionist multiple filters with the same window sizes move toward the output of the last layer. We used two ResNet blocks to learn the local features of the word vectors. A ResNet block is shown in Figure 2. Three convolutional layers exist in one block, and each layer is followed by a BatchNormalization and an ReLU activation.

Following He et al.'s work [16], we defined a building block as follows:

(1) $$\begin{array}{c} y=F\left(x,\left\{w_{i}\right\}\right)+x, \end{array}$$

where *x* and *y* are the input and output vectors of one ResNet block, and the final output of the ResNet layer is {*h*_1_, *h*_2_,…, *h*_*l*_} after the activation of ReLU.

We added the attention mechanism to the ResNet layer to capture the crucial components of the high-level semantic. For all the states {*h*_1_, *h*_2_,…, *h*_*l*_}, we define

(2) $$\begin{array}{c} u_{i}^{t}=d_{t}Wh_{i},\\ a_{i}^{t}=\text{softmax}\left(u_{i}^{t}\right),\\ c^{t}=\sum_{i}^{l}a_{i}^{t}h_{i}, \end{array}$$

where *c*^*t*^ is the encoded state calculated by the weighted sum of {*h*_1_, *h*_2_,…, *h*_*l*_} at time step *t* and *a*_*i*_^*t*^ is the weight of *h*_*i*_. *W* ∈ *R*^*d*×*d*^ and *d*_*t*_ ∈ *R*^*d*^ are used to transform *h*_*i*_ into a scalar. As shown in Figure 1, the model multiplies the outputs of the attention mechanism and ResNet and sends outputs to the next layer.

### 3.3. BiLSTM Module

LSTM has been proposed to overcome the gradient vanishing problem of RNN [7]. BiLSTM involves duplicating the first recurrent layer in the network such that two layers exist side-by-side, i.e., the as-is input sequence as the input to the first layer, and providing a reversed copy of the input sequence to the second layer [25]. The benefit of using the bidirectional network can be demonstrated by the sample sentence, “It is raining outside. I want to XXX for the whole day.” On the basis of “raining” we can predict that “XXX” may be “rest,” “sleep,” or “eat hotpot.” However, the follow-up phrase “for the whole day” indicates that “eat hotpot” is unsuitable. Moreover, unidirectional LSTM learns the knowledge only from one direction, but BiLSTM can learn the information from the whole context.

**Figure 3:**
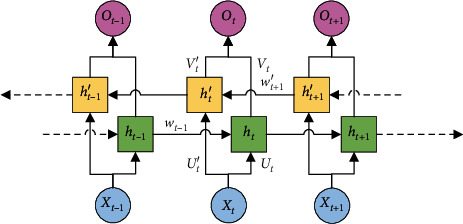
The framework of BiLSTM.

The BiLSTM framework is shown in Figure 3. The input is matrix *X* while the output is matrix *O*. *X* and *O* have the same sizes.

The sequence {*h*_1_, *h*_2_,…, *h*_*l*_} is the output of the forward-layer memory cell, whereas the sequence {*h*_1_′, *h*_2_′,…, *h*_*l*_′} is the output of the backward-layer memory cell. The detailed operation of BiLSTM can be defined as follows:

(3) $$\begin{array}{c} h_{t}=f\left(U_{t}x_{t}+W_{t-1}h_{t-1}\right), \end{array}$$

(4) $$\begin{array}{c} h_{t}^{\prime }=f{\left(U_{t}^{\prime }x_{t}+W_{t+1}^{\prime }h_{t+1}\right)}^{\prime }, \end{array}$$

(5) $$\begin{array}{c} o_{t}=g\left(V_{t}h_{t}+V_{t}^{\prime }h_{t}^{\prime }\right), \end{array}$$

where *U*_*i*_, *W*_*i*_, and *V*_*i*_ are the weight matrices of the network and *f*(·) and *g*(·) are nonlinear activation functions. At each time step *t*, the output *O*_*t*_ is computed on basis of the forward-layer state *h*_*t*_ and the backward-layer state *h*_*t*_′.

### 3.4. Improved Focal Loss and Output Layer

The output layer is the softmax classification [26]. The output size is the number of text classes, and the conditional probability value of each type is obtained by equation (6), where the softmax is a nonlinear activation to achieve probability normalization.

(6) $$\begin{array}{c} p_{i}=\frac{\exp\left(y_{i}\right)}{\sum_{j}\exp\left(y_{j}\right)}, \end{array}$$

where *p*_*i*_ (value of softmax) denotes the probability that the features reflect the class *i*, {*y*_1_, *y*_2_,…, *y*_*N*_} represents the output, and *i* and *j* ∈ {1,2,…, *N*}, where *N* is the number of classes.

The focal loss function is applied to solve the class imbalance instead of the cross entropy [14]. In order to increase the weight of small, we improved the focal loss by adding Gaussian weight. The less the number of samples in a certain class is, the greater the Gaussian weight is and the more the attention is paid by the model. The improved loss function is denoted as follows:

(7) $$\begin{array}{c} L_{\text{fl}}\left(p_{i}\right)=-\alpha_{i}{\left(1-p_{i}\right)}^{\gamma }\log\left(p_{i}\right), \end{array}$$

(8) $$\begin{array}{c} \alpha_{i}=\alpha +\beta \frac{1}{\sigma \sqrt{2\pi }}\exp\left(-\frac{c_{i}^{2}}{2\sigma^{2}}\right), \end{array}$$

where *α*_*i*_ is a weighting factor and *c*_*i*_ is the count of each class. The focal loss adds a modulating factor (1 − *p*_*i*_)^*γ*^ to the cross entropy loss with parameter *γ* ≥ 0. When *γ*=0, focal loss is equivalent to cross entropy, and as *γ* is increased, *α* should be decreased. In the range of 0 to 1, the smaller *α* is, the smaller the negative sample (the classes which have the large quantity) weight is. We improved *α*_*i*_ with a Gauss part by adding the weight of less numerous classes. In Lin et al.'s work [13], when *γ*= 2 and *α*= 0.25, the model worked best. We follow Lin's work and apply the same parameter setting. The parameters *β* and *σ* control the weight of every loss of classes and make sure *α*_*i*_ ∈ [0,1].

## 4. Experiments

### 4.1. Dataset

The dataset used in this study comprises criminal case description discourses from China judgements online. This dataset [27] contains 154,592 documents for training, 17,131 documents for validation, and 34,720 documents for testing. There are 202 types of events, and the first 5 types in terms of the largest and the smallest numbers are shown in Table 1.

Table 1 shows that the distribution of event types is seriously imbalanced. The number of maximum class is several hundred times larger than the number of minimum class in the training set.

There are three other datasets that we applied for model evaluation.

  SogouCA dataset contains 1,920,000 Chinese news documents from news websites collected by Sogou incorporation, and there are 18 classes of news. We use 80% of the data for training and 20% for testing.
  Chinese Scientific Literature (CSL) Dataset contains 31,489 Chinese scientific literatures which can be divided into 34 classes. We randomly select 80%/20% data as training and testing sets.
  AG's corpus of news articles from http://groups.di.unipi.it/∼gulli/AG_corpus_of_news_articles.html contains English news articles which have been gathered from more than 2000 news sources. There are 120,000 articles for training and 7,600 for testing in this dataset.

### 4.2. Metric

In order to evaluate the performance of our method, we computed the macro-average precision, recall, and geometric mean (G-mean) value [28]. F1 score is one of the most commonly used metrics of classifiers. However, comparing with F1 score, G-mean value can account for class imbalance properly. Because when one class is completely missed by the classifier, G-mean value of this class goes to zero. Thus, G-mean can clearly indicate the effect of the classifier on the problem of class imbalance. For a class *k*, let TP_*k*_(FP_*k*_) be the number of instances that are identified correctly (incorrectly) as positive ones and TN_*k*_(FN_*k*_) be the number of instances which are classified correctly (incorrectly) as negative ones. We can compute the macro-average precision Pre, recall Rec, and G-mean GM as in equations (9)–(11).

(9) $$\begin{array}{c} \text{Pre}=\frac{1}{N}\sum_{k=1}^{N}\frac{{\text{TP}}_{k}}{{\text{TP}}_{k}+{\text{FP}}_{k}}, \end{array}$$

(10) $$\begin{array}{c} \text{Rec}=\frac{1}{N}\sum_{k=1}^{N}\frac{{\text{TP}}_{k}}{{\text{TP}}_{k}+{\text{FN}}_{k}}, \end{array}$$

(11) $$\begin{array}{c} \text{GM}={\left(\prod_{k=1}^{N}\frac{{\text{TP}}_{k}}{{\text{TP}}_{k}+{\text{FN}}_{k}}\right)}^{\left(1/N\right)}, \end{array}$$

where *N* is the total number of classes.

### 4.3. Baselines

We benchmarked the following baseline methods for text semantic classification, which achieved good results in text classification:

- CNN: multilabel text classification model with convolutional layer of multiple filter sizes, max-pooling layer, and improved loss function proposed by Lin et al. [5].
- SR-LSTM: a supervised sentiment classification model learning sentences and document representations proposed by Huang et al. [14].
- BiLSTM + attention: extreme multilabel text classification model based on BiLSTM and attention mechanism proposed by You et al. [9].
- AC-BiLSTM: a model combining the strengths of CNN, RNN, and attention mechanism for text semantic extraction and classification proposed by Liu and Guo [11].
- ResNet + BiLSTM + attention + oversampling: the same basic architecture with our CRAFL model, using oversampling to solve class imbalance instead of focal loss based on the work of Li et al. [19].

### 4.4. Hyperparameter Setting

In order to optimize the model, we applied grid search combined with manual parameter adjustment to select the hyperparameter values. We set the hyperparameters as shown in Table 2. For quantitative factors, we reported the experiment results of different hyperparameter values in Section 5.3, and the considered admitted ranges were based on previous literature findings.

As shown in Table 2, we initialized word embedding layer with 300-dimensional Word2vec-trained word embedding layer. The kernel size and filters of all CNN layers are 3 and 512.

We trained our model by using Adam with gradient clipping. Adam designs an independent adaptive learning rate for different parameters by calculating the estimation of the first-order and the second-order moments of the gradient [29]. The dropout layer selects data randomly to guard against overfitting and renders the model to be much more robust [30]. The dimensionality of word vectors and the dropout value can affect time efficiency. Therefore, reasonable input dimensionality and dropout are necessary for modeling. We regulated our network with a dropout rate of 0.5 before the output layer, and the batch size is set to 32. The initialized learning rate is set to 0.001.

We used the same parameter settings as in the literatures of baseline models. For other parameters not described in the literatures, the settings are the same as our model.

## 5. Results and Discussion

### 5.1. Overall Performance

Table 3 shows the results achieved on the long discourse dataset. To avoid zeroing, when G-mean value of a class is 0, we set it to 0.001. In Table 3, CRAFL outperforms the baselines and can offer relative improvements of 23.8% compared with CNN and 20.3% and 18.1% relative improvements compared with SR-LSTM and BiLSTM + attention in terms of the value of G-mean. CRAFL also outperforms AC-BiLSTM even if they have similar structures. Moreover, the problem of class imbalance can be addressed by the improved focal loss function, and its effect is better than that by the oversampling approach.

A comparison of the methods based on CNN and LSTM showed that BiLSTM networks can achieve better results. Thus, for the semantic classification of long discourse data, LSTM is highly suitable for sequence data processing, and bidirectional information of discourse should be learned. As shown in Table 3, CRAFL performs better than the single CNN or LSTM neural network. This finding indicates that the CNN layer can extract preliminary features for the RNN layer to obtain good results.

The effects of the models with attention mechanism are also relatively good. These finding are consistent with the conclusions of many studies that the attention mechanism can focus on important information and improve the learning results [9–12, 17, 18].

The recall of the models is unstable because of the uneven input data. The recalls of models are usually unsatisfactory without the measures to deal with data imbalance. The results in Table 3 show that oversampling and improved loss function have a good effect on imbalanced data. The improved focal loss function can obtain better result than oversampling.

We also tested our method on other datasets and compared its results with the baselines in terms of the G-mean value.

As shown on Table 4, our model shows its advantages in these three datasets. For Sogou CA, all the methods have better performance because the data imbalance problem of this dataset is not serious. For English training data, our model also outperforms other methods because of its excellent understanding of English semantics.

### 5.2. Further Identification

In order to analyze the performance of our model, we also report the confusion matrix of the 5 largest and 5 smallest classes in Table 5.

From Table 5, obviously, the class labeled 185 is missed by the model. In the 5 smallest classes, the performance of the model in the other 4 classes is acceptable. For the 5 largest classes, the model shows its excellent performance. Above all, the model performs well in most large and small classes; however, it also ignores some small classes.

**Figure 4:**
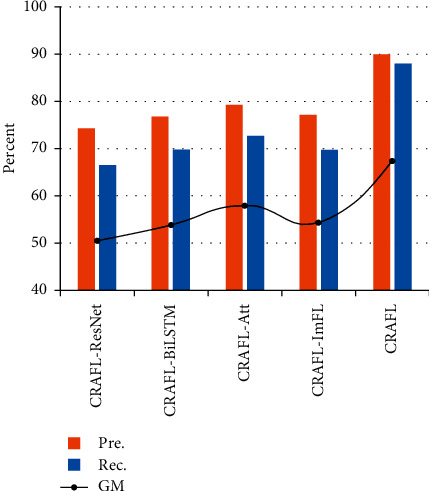
The effects of different model architectures.

In order to further study the role of each part of the model structure, we conduct experiments on some structures of CRAFL in Table 6 and Figure 4. The models depicted in Figure 4 are as follows: CRAFL-ResNet (the same architecture as CRAFL but by using CNN instead of ResNet), CRAFL-BiLSTM (the same architecture as CRAFL but by removing BiLSTM layer), CRAFL-Att (the same architecture as CRAFL but by removing attention mechanism), CRAFL-ImFL (the same architecture as CRAFL but by using the cross entropy loss), and CRAFL.

From Table 6 and Figure 4, it can be seen that ResNet can achieve better results than CNN for the long discourse dataset. Thus, ResNet is more suitable than some other previous CNN-based networks before the BiLSTM layer for the text semantic classification task. The role of the BiLSTM layer is the semantic feature extraction of the discourse. When this layer is removed, the effect of feature extraction will be significantly reduced. Moreover, the improvement of attention mechanism to the overall performance of the model shows that attention mechanism plays a role in the weighted calculation of word vector features.

The performance of the improved focal loss function is also superior to those of the cross entropy loss function and the standard focal loss. It demonstrates that focal loss can effectively solve the problem of class imbalance, and our introduced improvement can improve performance.

The oversized network can cause overfitting when the dataset is not large enough. This research used four CNN layers and only one BiLSTM layer to extract the features before handing them to the output layer. Furthermore, a specific structure is needed by particular task. For example, as presented in Table 2, AC-BiLSTM is more complex than BiLSTM + attention, but its effect is not obvious. This method is suitable for sentiment classification tasks with relatively few classes.

### 5.3. Other Observations

We improved focal loss function with adding a Gaussian weight. *β* and *σ* in equation (8) control the weights of classes. The largest class in training set has a total of 10,051 samples. Thus, we changed *σ* from 1000 to 5000, and the results of experiments are illustrated in Table 7.

From Table 7, it can be seen that when the value of *σ* ranges from 1000 to 5000, its influence on the results is not obvious because for the classes with large sample size, the change of *σ* has little effect on their weights.

We analyzed the word embedding dimension in range of [20, 500], and results are reported in Table 8.

As shown in Table 8, as the word embedding dimension increases, a great improvement was given. However, for more than 300 dimensions, adding other dimensions does not give a significant improvement. Therefore, also in accordance with most of the previous literature works [31], the value 300 was chosen as optimal for the word embedding dimension.

As one of the most important hyperparameters defining the learning procedure, batch size is analyzed by considering the following values: {8, 32, 128, 512}. Results are reported in Table 9.

From the results of Table 9, the smaller the batch size was, the better the model representation was obtained. Moreover, the influence of batch size was not significant between 4 and 32. However, smaller batch sizes cause longer training time. Thus, batch=32 was chosen [32].

The dropout was analyzed as well, and we varied dropout in the range (0, 1), in correspondence to the following representative levels: dropout=0.1, 0.3, 0.5, 0.9. Results are reported in Table 10.

From Table 10, when the value of dropout is close to 1, the performance decreases obviously. And when dropout=0.1, the effect of dropout is not fully demonstrated. Moreover, when the amount of data is relatively large, dropout can play a great role [33]. Therefore, dropout=0.5 was chosen.

The performance of the model is the best when the convolution kernel is adjusted slightly. During the training process, the highest accuracy was obtained when the convolution kernel was 3*∗*1, which was close to the window sizes of current words and predictive words.

## 6. Conclusions

In this work, we proposed the CRAFL model for the text semantic classification of Chinese discourse. For the first time, the problems of sparse long discourses classification and class imbalance have been addressed by a ResNet and BiLSTM-based model with attention mechanism and improved focal loss. The experiments show that CRAFL can achieve state-of-the-art performance on a long discourse dataset. Thus, it demonstrates that the combination of ResNet and BiLSTM is suitable for long discourse semantic extraction, and our improvement of focal loss function can solve the problem of data imbalance. However, when the dataset is extremely imbalanced, our model ignores some classes with small amount of data. Thus, for the dataset with especially small classes, the problem of data imbalance is difficult to solve completely.

Future studies will focus on discourse relationship recognition by exploring ways to utilize the implicit text semantic of long discourse. We plan to explore other sequence learning models for semantic feature extraction of discourse and further evaluate our approach in other application domains.

## Figures and Tables

**Table 1:** Largest and smallest types of events.

Type of event	Label	Count
Larceny	190	10,051
Smuggling, trafficking, transporting, making drugs	73	8,872
Intentional assault	191	6,377
Robbery	19	5,020
Fraud	98	3,536
Destruction of vehicles	134	19
Relending at high interest rate	67	18
Reselling cultural relics	79	17
Abuse of guardians	105	17
Smuggling	185	16

**Table 2:** Hyperparameter setting of our model.

Hyperparameters	Symbol	Value
Word embedding dimension	*D*_*w*_	300
Kernel size of CNN	*S*_*k*_	3
Number of filters	*N*_*f*_	512
Activation function	*f*	ReLU
Weights updating rule	Optimizer	Adam
Dropout	Dropout	0.5
Batch size	Batch	32
Learning rate	*l*_*r*_	0.001

**Table 3:** Results of experiments compared with baselines.

Model	Pre. (%)	Rec. (%)	GM (%)
CNN [5]	70.2	50.8	43.6
SR-LSTM [14]	73.8	67.0	47.1
BiLSTM + attention [9]	77.1	69.7	49.3
AC-BiLSTM [11]	76.1	70.9	51.8
ResNet + BiLSTM + attention + oversampling [19]	81.6	77.8	59.2
CRAFL	90.0	88.0	67.4

**Table 4:** Results of experiments compared with baselines.

Model	Sogou CA	CSL	AG's
CNN [5]	57.4	52.7	53.7
SR-LSTM [14]	63.5	55.1	58.3
BiLSTM + attention [9]	65.3	59.8	62.4
AC-BiLSTM [11]	67.0	63.9	64.0
ResNet + BiLSTM + attention + oversampling [19]	70.8	65.4	69.8
CRAFL	75.7	70.2	75.1

**Table 5:** Confusion matrix of the largest and smallest events in testing.

Label	TP	FP	FN	TN	Pre. (%)	Rec. (%)	GM (%)
190	1769	136	79	32736	92.9	95.7	97.8
73	1590	114	49	32967	93.2	97.0	98.5
191	1122	104	64	33430	91.5	94.6	97.3
19	801	78	90	33751	91.1	89.9	94.8
98	583	81	70	33986	87.8	89.3	94.5
134	2	1	2	34715	66.7	50.0	70.7
67	67	18	2	34715	66.7	50.0	70.7
79	3	0	1	34716	100.0	75.0	86.6
105	1	0	3	34716	100.0	25.0	50.0
185	0	3	2	34715	0	0	0

**Table 6:** Results of experiments compared with baselines.

Model	Pre. (%)	Rec. (%)	GM (%)
CRAFL-ResNet	74.2	66.4	50.5
CRAFL-BiLSTM	76.8	79.7	53.8
CRAFL-Att	79.3	72.6	57.9
CRAFL-ImFL	77.1	69.7	54.3
CRAFL	90.0	88.0	67.4

**Table 7:** G-mean value obtained by varying *σ* (*β*=100).

*σ*	1000	2000	3350	4000	5000

GM (%)	66.0	66.0	67.4	66.5	65.3

**Table 8:** G-mean value obtained by different embedding dimensions.

*D*_*w*_^1^	20	100	300	500

GM (%)	57.8	61.0	67.4	67.5

^1^Other settings were *S*_*k*_=3, *N*_*f*_=512, *f*=ReLu, optimizer=Adam, dropout=0.5, batch=32, and *l*_*r*_=0.001.

**Table 9:** G-mean value obtained by different batch sizes.

Batch^1^	4	32	128	512

GM (%)	67.5	67.4	63.7	60.0

^1^Other settings were *S*_*k*_=3, *N*_*f*_=512, *f*=ReLu, optimizer=Adam, dropout=0.5, *D*_*w*_=300, and *l*_*r*_=0.001.

**Table 10:** G-mean value obtained by different dropout.

Dropout^1^	4	32	128	512

GM (%)	67.5	67.4	67.4	64.8

^1^Other settings were *S*_*k*_=3, *N*_*f*_=512, *f*=ReLu, optimizer=Adam, batch=32, *D*_*w*_=300, and *l*_*r*_=0.001.

## Data Availability

The XML data used to support the findings of this study are included within the article. Previously reported XML data are available at https://github.com/china-ai-law-challenge/CAIL2018. These prior studies (and datasets) are cited at relevant places within the text as references [27].
